# Dietary supplements in polycystic ovary syndrome–current evidence

**DOI:** 10.3389/fendo.2024.1456571

**Published:** 2024-09-27

**Authors:** Ya Han, Ye Hou, Qimao Han, Xingxing Yuan, Lu Chen

**Affiliations:** ^1^ School of Graduate Studies, Heilongjiang University of Chinese Medicine, Harbin, China; ^2^ Department of Integrative Chinese and Western Medicine, Xin Cheng Health Care Hospital, Daqing, China; ^3^ Department of Rheumatology, First Affiliated Hospital of Heilongjiang University of Chinese Medicine, Harbin, China; ^4^ Department of Gastroenterology, Heilongjiang Academy of Traditional Chinese Medicine, Harbin, China; ^5^ Department of Gynecology II, First Affiliated Hospital of Heilongjiang University of Chinese Medicine, Harbin, China

**Keywords:** PCOS, infertility, dietary supplement, metabolic issues, treatment options

## Abstract

Polycystic ovary syndrome (PCOS) is the most prevalent endocrine disorder in women of reproductive age, and presents a significant challenge to the global population. This review provides comprehensive evidence of interventions, including food and dietary supplements, aimed at reversing PCOS and improving fertility outcomes. Various dietary supplements are known to cause metabolic changes and hormonal regulation and have a potential impact on increasing pregnancy rates. Although some biochemical alterations have been observed, these metabolic changes do not directly reverse the disorder. Moreover, the lack of sufficient evidence does not convince clinicians to standardize dietary supplements as alternatives to medical or pharmacological interventions. This calls for a study of women with PCOS taking dietary supplements. In addition, unbiased studies of combinations of treatment options for supplements, including large cohort clinical trials, will lead to evidence-based medicine.

## 1 Introduction

Polycystic ovarian syndrome (PCOS) is the most prevalent and extensively researched endocrine disorder affecting women of reproductive age. It affects up to 18% of women of reproductive age and is characterized by hyperandrogenism, chronic anovulation, and irregular menstrual cycles. In addition, PCOS has implications beyond reproductive issues, including infertility, miscarriage, and neonatal and pregnancy complications, but also increases the risk of psychological disorders including depression, cardiovascular disease including coronary atherosclerotic heart disease, and metabolic disorders including diabetes (1, 2). Reduced insulin sensitivity (IS) in women with obesity contributes to hyperinsulinemia and may lead to PCOS (3). PCOS-related infertility is attributed to oligo-amenorrhea and hyperandrogenic anovulation. Notably, anovulation is associated with arrested antral follicular development and low levels of follicle-stimulating hormone during the final stages of maturation (4). PCOS significantly impairs reproductive health and is strongly associated with obesity. Additionally, obesity-related inflammation affects the ovaries and induces physiological changes due to IS impairment. In pathological mechanistic studies of PCOS, insulin resistance (IR) is a central etiological factor independent of comorbidities such as obesity. Excessive ovarian androgen production is stimulated by subsequent hyperinsulinemia (5).

Many women of reproductive age have a strong desire to become mothers, which is deeply influenced by cultural and religious values in many human communities. Therefore, comprehensive Food and Drug Administration (FDA) approvals are needed for drugs currently under trial. Non-pharmacological therapies, such as food, dietary supplements, and herbal medicines, are frequently chosen over pharmacological treatments despite their potential adverse effects. Mechanistic studies based on biochemistry are needed to distinguish these supplements and understand their potential in combination therapy (6). For example, women with PCOS have a high prevalence of vitamin D deficiency, leading to the high preference for dietary supplementation (7). In addition, antioxidant supplements have been shown to have several beneficial effects in the treatment of PCOS as they improve IR, fasting blood insulin, and glucose levels in women with PCOS (8, 9).

One urgent priority in medical and clinical research is the need for optimal treatment options. Considering the sensitive nature of reproductive health cases, reliance on current literature is needed to establish an unbiased literature summary. To this end, this mini-review aimed to examine the most recent publications using the search terms ‘PCOS and dietary supplements.’ PubMed, Web of Science, Embase, and Google Scholar were searched. After removing duplicates by title scanning, 69 articles were deemed suitable for analysis. The selected articles were critically and qualitatively assessed to provide reports and graphical descriptions that offered valuable insights to researchers. This review focuses on supplements such as vitamin D, Myo-inositol, selenium, probiotics, or synbiotics. Although not exhaustive, this qualitative mini-review serves as a valuable resource for reproductive health researchers conducting advanced studies.

## 2 Supplements for PCOS

Several pharmaceutical treatments have been suggested for the treatment of PCOS. Oral contraceptives are the most common choice for PCOS treatment; however, they do not promote natural ovulation (10). Clomiphene and letrozole are widely used to induce ovulation; however, they have adverse effects and low adherence to long-term medication (11). Metformin and thiazolidinediones are used to improve IR in patients with PCOS. However, discrepancies in their clinical results and increasing side effects, especially in non-obese, non-IR patients, have raised doubts regarding their efficacy.

**Figure 1:**
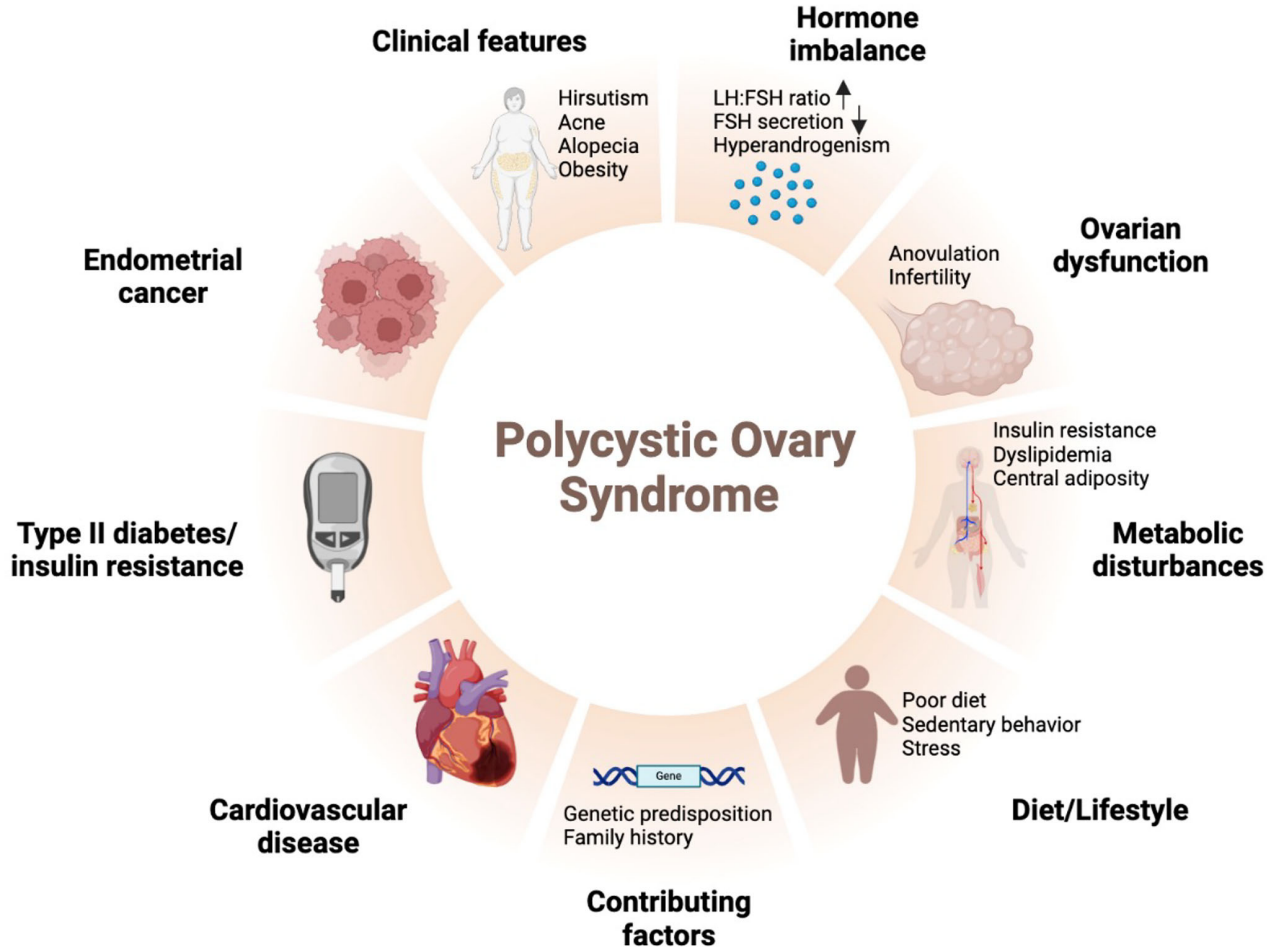
Schematic diagram of polycystic ovary syndrome.

Personalized dietary therapy and weight loss, if required, are first-line treatments for young women with PCOS and obesity (12). In women with obesity, weight loss of at least 5% can improve hyperandrogenism, IR, fertility, and menstrual function (13). Dietary therapy prevents infertility, restores normal body mass, improves physiology, and preserves ovarian health. Methyl donor supplements, antioxidants, and correct protein intake can also reduce toxic oxidants and protect egg maturation (14). Natural molecules derived from herbal medicines and nutritional supplements, such as vitamin D, curcumin, Coenzyme Q_10_ (CoQ_10_), N-acetylcysteine, and inositol, have been shown to play a therapeutic role in ameliorating inflammation and IS, restoring ovarian function, maintaining regular hormonal balance, and normalizing the menstrual cycle, and have fewer side effects with clinical significance (15–17) (**Figure 1**).

## 3 Vitamin D and vitamin E as supplements in PCOS

Vitamin D improves insulin metabolism, increases total antioxidant capacity (TAC), reduces hirsutism, and reduces C-reactive protein (CRP) and total serum testosterone levels (18). It regulates ovulation dysfunction, hyperandrogenism, and IR in the ovaries through the 1 α-hydroxylase (CYP27B1) and vitamin D receptor (VDR) genes. Due to the anti-inflammatory and immunomodulatory properties of vitamin D, vitamin D deficiency may lead to the pathogenesis of endometriosis, which is another important cause of female infertility besides PCOS (19). In humans, 25 OH-D is positively correlated with anti-Müllerian hormone (AMH) and appropriate levels of vitamin D. Normalization of abnormal AMH levels indicates improves folliculogenesis and protects against endometriosis (20). Vitamin D alters serum AMH signaling, progesterone release, and follicle-stimulating hormone (FSH) sensitivity in *in vitro* studies (21).

To determine the potential effects of vitamin D on reproductive health, serum AMH levels are insufficient as a biomarker. For instance, the relationship between vitamin D supplementation and AMH levels differs between women with PCOS and those without PCOS, indicating a correlation dependent on ovulatory status (22). Another contrasting study revealed that vitamin D supplementation in women with PCOS reduced FSH, luteinizing hormone (LH), and androgen levels and decreased early miscarriage rates (23). Moreover, vitamin D regulates metabolism by reducing malondialdehyde (MDA) and high-sensitivity CRP levels while increasing TAC (24). However, vitamin D supplementation has insufficient evidence regarding reversing sexual dysfunction and depression in women with PCOS, despite its role in regulating the menstrual cycle and ovulation. Moreover, the molecular properties of anti-sperm antibodies binding to vitamin D-binding protein (VDBP) raise questions regarding their role in implantation (25). In addition, high doses of vitamin D may have toxic effects, and routine screening should be conducted for patients with PCOS before recommending any supplements (26).

A noteworthy sister supplement to vitamin D is vitamin E, which has been shown to improve glucose and lipid metabolism and other androgenic-related biomarkers in women with PCOS (27). Vitamin E supplementation has been associated with reduced homeostatic model assessment of insulin resistance (HOMA-IR) levels, LH, and testosterone concentrations, and increased FSH and progesterone concentrations. In addition, total glyceride (TG) and low-density lipoprotein cholesterol (LDL-C) levels were significantly improved in women with PCOS taking vitamin E supplements (28).

## 4 Myo-inositol supplementation in PCOS

Myo-inositol and D-chiro-inositol are the two most clinically important isoforms of inositol; as secondary messengers of insulin, an imbalance between them is key to IR (29). Myo-inositol is a natural molecule present in fruits and vegetables that participates in FSH signaling, which orchestrates ovulation (30). Oral supplementation with myoinositol has been shown to improve hyperandrogenism and menstrual cycles, and restore spontaneous ovulation in women with PCOS (31). Myo-inositol effectively reduced postprandial triglyceride (TC) levels, glutathione peroxidase (GSH-Px) activity, liver fat accumulation, and aspartate aminotransferase (AST) levels (32). Furthermore, accumulating evidence suggests that myo-inositol has a beneficial effect on folliculogenesis, with improved oocyte maturation and embryo quality in women with PCOS undergoing *in vitro* fertilization (IVF) (33). Despite the conflicting fertility outcomes, myo-inositol has been found to shorten the duration of controlled ovarian hyperstimulation during IVF procedures (34). However, physicians remain unconvinced of its effectiveness in improving clinical pregnancy and live birth rates (35). Therefore, large-scale clinical trials are needed to explore the potential benefits of myoinositol in women with PCOS.

## 5 Selenium supplementation in PCOS

Selenium has antioxidant and anti-inflammatory properties and is an essential trace element. Selenium supplementation can effectively reverse biochemical disorders and serve as a biochemical marker in women with PCOS. Selenium supplementation reduces fasting plasma glucose (FPG), cholesterol, insulin, and TC levels (36). Another study showed an improvement in TAC, but selenium supplementation showed no significant effects on total testosterone levels or body mass index (BMI). Considering its antioxidant properties that enhance follicle quality, selenium can be recommended for patients with PCOS undergoing IVF (37).

## 6 Probiotic, prebiotic, and synbiotic supplementation in PCOS

Recent research has revealed a significant correlation between gut microbiota composition and PCOS pathogenesis (38). Modulation of the gut microbiota improves lipid metabolism, hormonal profiles, and inflammatory indicators in adolescents with PCOS (39). Probiotics are live microorganisms that, when ingested in sufficient amounts, can confer health benefits by modulating the gut microbiota, inhibiting the growth of pathogenic organisms, and reducing gut inflammation and permeability (40). Typical bacterial species that respond to probiotics include *Bifidobacterium breve*, *Bifidobacterium longum*, *Streptococcus salivarius* subsp*. thermophilus*, and *Lactobacillus* species such as *L. acidophilus*, *L. casei* and *L. delbrueckii* (41). Probiotic supplementation has been associated with protective effects on fertility-related factors such as body weight normalization, BMI regulation, insulin control, and HOMA-IR improvement in women with PCOS (42). In addition, probiotic supplements can reduce FPG and testosterone levels, whereas synbiotics can reduce fasting blood insulin levels.

Probiotics are usually indigestible dietary carbohydrates that promote the growth and function of beneficial microorganisms in the gastrointestinal tract (43). Prebiotics have the potential to enhance host metabolism, decrease proinflammatory markers, and improve lipid profiles by stimulating the growth of beneficial bacteria, such as *Lactobacillus* and *Bifidobacterium*. Prebiotics have shown positive effects on anthropometric parameters, FPG, and CRP levels in women with PCOS (44).

Synbiotics are combinations of probiotics and prebiotics designed to enhance the survival of beneficial bacteria in the gut (45). Synbiotics have been demonstrated to reduce FPG levels and improve IS, IR, CRP, and total testosterone levels, leading to enhanced glucose homeostasis, hormonal balance, and inflammatory markers in women with PCOS (46).

Overall, improved hormonal indices such as the free androgen index (FAI) and sex hormone-binding globulin (SHBG) are possible with probiotics, prebiotics, and synbiotics, as well as the regulation of inflammatory markers such as nitrogen oxide (NO) and MDA (47). Moreover, the effects of probiotics and synbiotics on metabolic, inflammatory, and hormonal parameters led to improved fertility in women with PCOS (48).

## 7 Other supplements in PCOS

CoQ_10_, an antioxidant, has been shown to be effective in women with PCOS who underwent assisted reproductive technologies (ART) (49). It is considered safe and can reduce IR while increasing FSH and improving blood lipids (50). Flaxseed supplementation, which is rich in dietary fiber, phytoestrogens, lignans, and α-linolenic acid (ALA) have the potential to improve metabolic, anthropometric and hormonal parameters (51). However, further studies with larger cohorts are required to establish the clinical benefits in women with PCOS. Folic acid supplementation can reduce BMI, especially in patients with PCOS and high homocysteine levels (52). Although L-carnitine has been shown to improve BMI and LDL-C, TG, and TC levels in women with PCOS, it has not demonstrated positive effects in patients with PCOS-related infertility (53). Zn is a crucial trace element recognized as an insulin mimic that promotes adipogenesis and glucose uptake by isolated adipocytes in a manner similar to that of insulin. In women with PCOS, Zn supplementation has beneficial effects on numerous parameters, particularly those associated with insulin resistance and lipid balance (54). Choline supplementation improved ovarian function in a pig model. This opens new doors for studying its positive effects on ovarian phenotypes in humans (55).

The significant reduction in body weight and BMI associated with cinnamon demonstrates its effect on anthropometric indices in women with PCOS (56). Despite contrasting findings from animal model studies, cinnamon has been shown to improve IR in women with PCOS (57). Moreover, oral cinnamon supplementation improved the metabolic parameters (58). Decreases in TC, TG, LDL-C, body weight, BMI, and hip circumference were observed with carnitine supplementation. Although it does not affect glucose homeostasis, large clinical trials of carnitine in women with PCOS have shown promising results in terms of lipid and weight loss profiles (59). Increasing evidence suggests that estrogen supplementation positively affects the quality of immature oocytes for maturation in stimulated IVF cycles (60). Curcumin plays a pivotal role in glycemic control; however, it exerts minimal influence on most reproduction-associated metabolic processes (61). Curcumin effectively improves FBG, IS, IR, HDL-C, and TC levels in women with PCOS, with no significant impact on LDL-C and TG levels (62).

**Figure 2:**
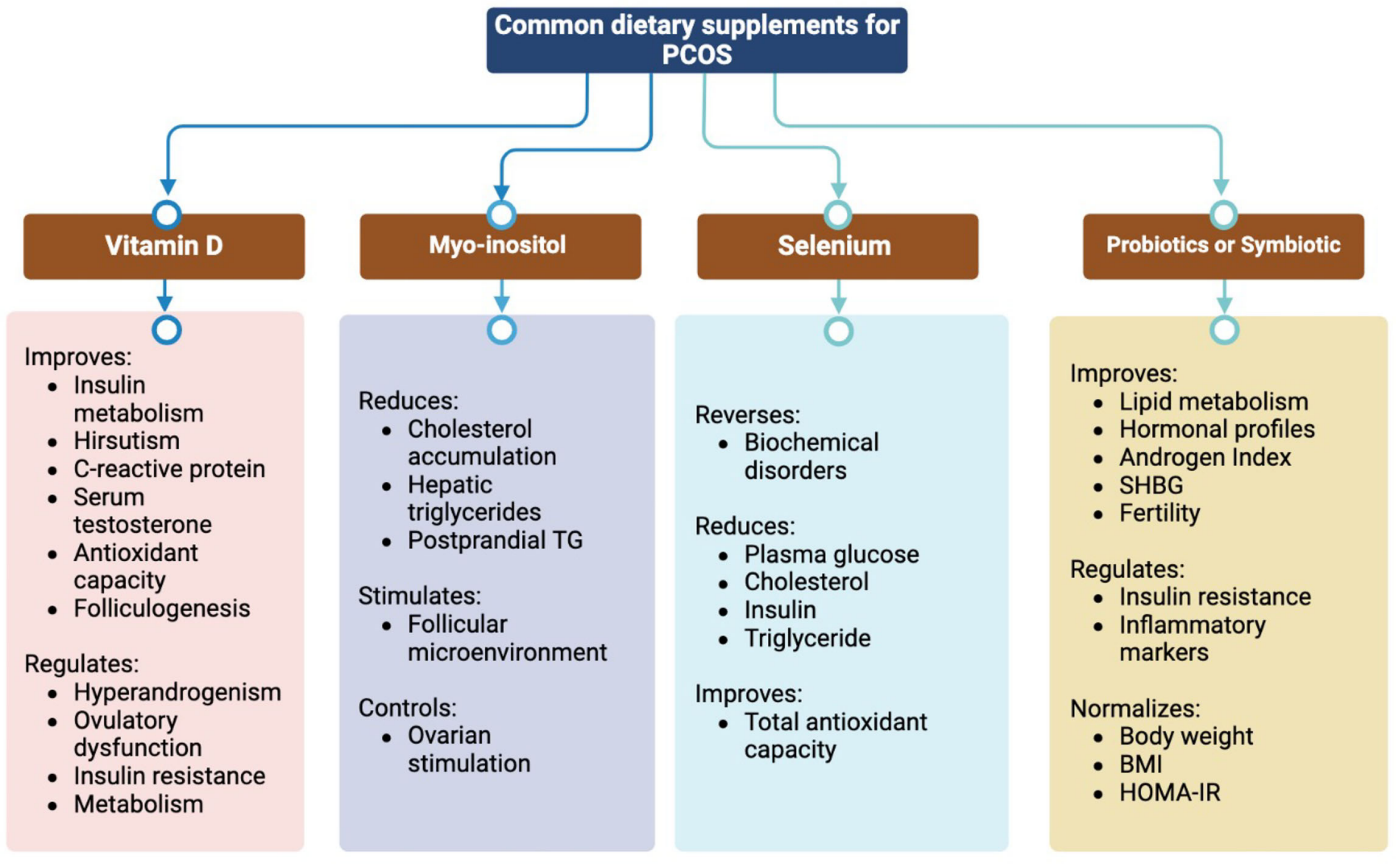
Effects of common dietary supplements on polycystic ovary syndrome.

Recently, the potential role of Cr supplementation has received increasing consideration in improving or preventing PCOS (63, 64). Although there are no beneficial effects on total testosterone, DHEAS, FSH, and LH levels, recent studies have suggested that Cr supplementation can lower BMI, free testosterone concentration, and fasting blood insulin (65). Except for DHEAS, omega-3 polyunsaturated fatty acids (omega-3 PUFAs) have shown very low significance as anti-androgen agents in women with PCOS (66). Omega-3 PUFAs improve HOMA-IR, insulin levels, TC, TG, very low-density lipopolysaccharide cholesterol (VLDL-C), LDL-C, and HDL-C. However, their effect on serum glucose levels remains unclear due to the limited number of studies (67). Omega-3 PUFAs reduce high-sensitivity CRP while increasing the adiponectin concentration, revealing their anti-inflammatory properties in women with PCOS (68) (**Figure 2**).

## 8 Recommendations for supplementations on PCOS

Reproductive health is a highly sensitive aspect of clinical decision-making, and it is essential for both gynecology and obstetrics experts to adhere to evidence-based recommendations. For instance, the modified Delphi process can help establish a consensus on micronutrient supplementation in women of reproductive age (69). In terms of clinical significance, personalized administration of probiotics with known strain types, dosage composition, defined impact on individual autochthonous microbiota, improved data on endocrine regulation, and hormonal values are necessary to determine conclusive clinical relevance in women with PCOS (70). Recommendations for supplementation in women with PCOS should consider a balanced diet and specific elements needed by the patient based on their comorbidities. Although these are ascribed to personalized medicine, an optimal dosage will minimize excessive consumption (71).

Multicenter and multi-country studies will provide insights into the genetic, environmental, and socioeconomic conditions that may have prevailed from childhood to adolescence in patients with PCOS (72). Moreover, a study on reproductive parental dietary supplements will answer questions regarding the epigenetic link between adolescents with PCOS and their parents (73). Notably, to achieve optimal management of PCOS, sex hormone assays must be valid and accurate. Laboratory errors and analytical interference should be carefully cross-checked, especially when test results contradict clinical characteristics. When evaluating serum estradiol levels, potential interferences such as heterophiles can be excluded using alternative immunoassay platforms (74).

Combination therapies using multiple supplements can have synergistic effects. Probiotics can not only reduce oxidative stress, hyperandrogenism, and inflammation, but also demonstrate a synergistic effect with vitamin D by enhancing VDR expression (75). Therefore, co-supplementation with probiotics and vitamin D has been shown to provide significant health benefits in terms of mental health, serum total testosterone, and oxidative stress parameters (76). In addition, the combination of probiotics with selenium or vitamin E and omega-3 polyunsaturated fatty acids is more effective in terms of mental health, hormonal profiles, and inflammation biomarkers (77). Considering the limited efficacy of omega-3 PUFAs in modulating hormone levels, the combination of vitamin E with omega-3 PUFAs or magnesium had an optimal effect on hormonal profiles, glycemic indices, HDL-C levels, and other biomarkers (78). Co-supplementation of magnesium, zinc, calcium, and vitamins D or E can improve IS and CRP levels, lipid profiles, and glucose metabolism in women with PCOS (79). Fertility treatment by reversing IR and administering inositol has been found to be the most effective option. The combination of myo-inositol and metformin demonstrated clear clinical benefits in women with PCOS undergoing induction cycles (80). However, the use of this combination in relation to the clinical outcome of ART is still under scrutiny because of limited evidence from clinical trials (81). While this review is qualitative in nature, quantitative studies are needed, such as meta-analyses or systematic reviews, that include a number of supplements with similar benefits. Such reviews should also recommends some data-driven information for to reproductive health researchers to establish the conditions for informed consent.

In conclusion, this review highlights the potential benefits of dietary supplements in improving metabolic, anthropometric, and hormonal parameters in women with PCOS, including those undergoing ART. Among the most promising supplements, long-term observational studies should be conducted to assess the potential toxic effects of vitamin D at different doses and administration durations. This review highlights the limited number of dietary supplements studied and the need for further research on their essential elements. In addition to large-scale clinical trials, emphasis has been placed on enhancing reproductive issues. Although probiotics, prebiotics, and synthetic agents have emerged as potential supplements, their role in PCOS remains poorly understood. Therefore, further clinical trials are necessary to validate their therapeutic efficacy.
